# Targeting Human Cytomegalovirus as a Novel Approach for Glioblastoma Treatment

**DOI:** 10.3390/pathogens14121291

**Published:** 2025-12-16

**Authors:** Thelma Flores, Eloïse Delpierre, Ghislain Male, Claire Gourin, Sébastien Hantz, Alexia Damour, Gaëtan Ligat

**Affiliations:** 1 Institut Toulousain des Maladies Infectieuses et Inflammatoires (Infinity), Université de Toulouse, INSERM, CNRS, UT, Hôpital Purpan-BP 3028, 1 Place du Dr Baylac, 31024 Toulouse, France; 2 INSERM, RESINFIT, U1092, Université de Limoges, 2 rue du Dr Marcland, 87025 Limoges, France

**Keywords:** human cytomegalovirus, glioblastoma, treatments, targets

## Abstract

Glioblastoma (GB) is a highly aggressive brain tumor with a very poor prognosis. Treatment usually consists of surgery, followed by radiotherapy and chemotherapy, but the prognosis remains poor due to its resistance to therapies and a high recurrence rate. Multiple studies have reported the presence of human cytomegalovirus (HCMV) proteins and/or nucleic acids in GB tissues, suggesting its possible implication. These findings have led to the hypothesis that HCMV may contribute to tumor progression, immune evasion, angiogenesis, and resistance to therapy. Clinical trials using anti-HCMV therapies have shown promising preliminary results, indicating a potential therapeutic benefit. This review aims to provide a comprehensive overview of the current evidence linking HCMV to GB and the therapeutic implications. A deeper understanding of this complex interaction could unveil novel strategies for GB treatment.

## 1. Understanding Glioblastoma: Biology and Clinical Implications

Glioblastoma (GB) is the most common and aggressive primary brain tumor in adults. It is classified as a grade IV astrocytoma and is characterized by rapid proliferation, necrosis, microvascular proliferation, and diffuse infiltration. GB arises either de novo (primary GB) or through progression from lower-grade gliomas (secondary GB or astrocytoma, IDH-mutant), with each subtype displaying distinct molecular profiles [1]. Several genetic and environmental factors have been associated with its development. Primary GB is typically driven by early genomic alterations such as epidermal growth factor receptor (*EGF-R*) amplification, telomerase promoter (*TERT*) mutations, phosphatase and tensin homolog (PTEN) loss, and chromosomal instability, whereas secondary GB or astrocytoma, IDH-mutant often evolves through a stepwise accumulation of mutations, most characteristically *IDH1/2* mutations and ATP-dependent helicase ATRX loss. Epigenetically, GB frequently exhibits widespread DNA methylation changes, including the well-described O^6^-methylguanine-DNA methyltransferase (MGMT) promoter methylation, which also influences treatment response [2,3]. Large-scale transcriptomic studies have defined molecular subtypes—classical, mesenchymal, and proneural—reflecting distinct oncogenic programs and cells of origin [1]. Beyond genetics, proposed risk factors include prior exposure to therapeutic ionizing radiation, though lifestyle and environmental risks remain poorly supported. Together, these findings illustrate that GB pathogenesis results from a complex interplay of genetic, epigenetic, and microenvironmental drivers rather than a single causative factor. Clinically, diagnosis relies on magnetic resonance imaging (MRI) and histopathology. Imaging typically shows a ring-enhancing lesion with central necrosis, perilesional edema, and mass effect. The standard treatment combines surgical resection, radiotherapy (60 Gy over 6 weeks), and temozolomide chemotherapy [4,5]. Even so, the prognosis remains poor, with a median survival of 12–15 months and an almost inevitable recurrence. Current research focuses on overcoming resistance mechanisms and the immunosuppressive tumor microenvironment (TME).

## 2. Human Cytomegalovirus: A Multifaceted Pathogen

Human cytomegalovirus (HCMV) is a large, enveloped double-stranded DNA virus belonging to the *Betaherpesvirinae* subfamily. Its genome spans approximately 230 kilobases and encodes over 200 distinct proteins. Like other members of this subfamily, HCMV displays strict species specificity while retaining the ability to infect a wide range of cell types [6,7]. In immunocompetent individuals, primary HCMV infection is typically asymptomatic or may manifest as a mild, self-limited febrile illness. Although viremia is rapidly controlled by cell-mediated immunity, viral genomes persist in a latent form for the lifetime of the host. In contrast, viral reactivation or uncontrolled replication can result in severe, life-threatening complications in immunocompromised patients [8]. Moreover, HCMV is recognized as the leading infectious cause of congenital malformations worldwide, contributing to developmental delay, sensorineural hearing loss, and intrauterine or neonatal death in approximately 10–15% of infected cases [9]. Despite major advances in both diagnostic approaches and antiviral therapy, HCMV continues to represent a significant clinical issue [6,7].

## 3. The Link Between HCMV and GB

For the first time, HCMV was classified recently as possibly carcinogenic to humans (Group 2B) by the International Agency for Research on Cancer (IARC), with evidence in humans relating mainly to acute lymphoblastic leukemia in children [10,11]. While the presence of HCMV within tumors remains debated, a substantial body of evidence supports its detection in GB tissues. The pioneering work of Cobbs et al. first reported HCMV in 95% of malignant gliomas, a prevalence markedly higher than that observed in the general population [12]. Subsequent studies have highlighted the complex interplay between HCMV and GB, suggesting that the virus may modulate immune cell activity and reshape the TME. Recent evidence also indicates that HCMV can be reactivated in GB patients undergoing brain radiotherapy [13]. Moreover, antiviral drugs commonly used to combat HCMV infections, such as valganciclovir, have shown promising results in the treatment of GB, by slowing tumor progression and improving patient survival by over 40 months in some cases [14]. In line with these findings, anti-HCMV therapy could now appear as a promising strategy to halt GB progression.

## 4. Mechanistic Insight of the Dual Facets of HCMV in GB: Oncomodulation and Beyond

Even though HCMV was classified as possibly carcinogenic to humans, this classification was based on limited evidence on acute lymphoblastic leukemia (ALL) in children whose bloodspots showed HCMV DNA or whose mothers were HCMV IgM seropositive during pregnancy [11]. As for the evidence for the association with GB and its direct oncogenesis, it was deemed “inadequate”. Henceforth, further studies are needed to determine if HCMV plays a role in GB development.

**Figure 1:**
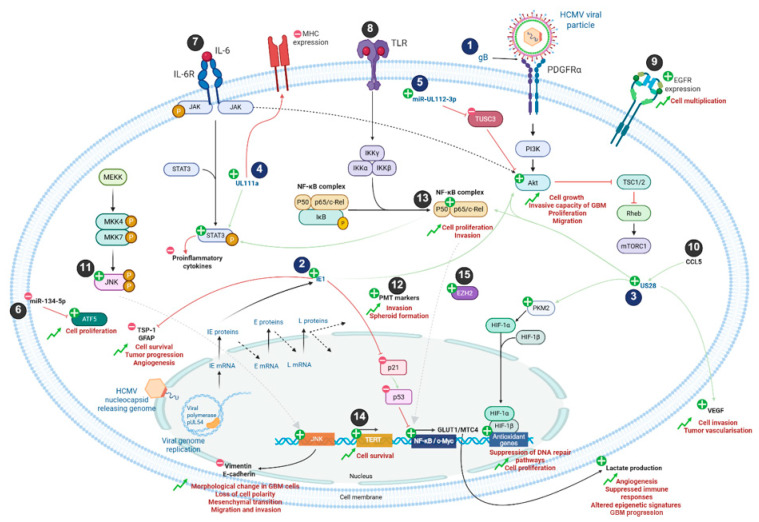
Mechanistic implications of HCMV infection in GB cells. Viral and cellular components are represented in blue and black, respectively. Plus and minus signs represent upregulation or downregulation of signaling pathways, gene expression and protein production, respectively. During GB formation, HCMV infection activates numerous cellular signaling pathways such as JNK, PI3K/Akt, NF-κB, PKM2/HIF and STAT3 through viral proteins including IE1, US28 and UL111a. In addition, the viral miR-UL112-3p is involved in the upregulation of the PI3K/Akt pathway. This results in increased proliferation, invasion, growth, and migration of GB cells, as well as reduced levels of pro-inflammatory cytokines. Upregulation of JNK gene products is responsible for decreased expression of vimentin and E-cadherin, leading to morphological changes and mesenchymal transition. Lactate production is also increased by the NF-κB pathway and promotes angiogenesis, suppresses immune responses, alters epigenetic signatures, and enhances GB progression. In addition, cellular markers such as ATF5, EZH2, or PMT are also overexpressed during GB and enable its formation. HCMV infection further assists in the establishment of the GB TME. Numbers 1 to 15 refer to Table 1 to enhance figure comprehension. IL: interleukin; MHC: major histocompatibility complex; TLR: Toll-like receptor; PDGFR: platelet-derived growth factor receptor; EGFR: epithelial growth factor receptor; VEGF: vascular endothelial growth factor; ATF5: activating transcription factor 5; PMT: proneural–mesenchymal transition; miR: miRNA.

Emerging evidence suggests that several mechanisms may contribute to HCMV playing a role in tumor progression (Figure 1 and Table 1). Glycoproteins present on the viral envelope interact with specific cell surface receptors—such as platelet-derived growth factor receptor alpha (PDGFRα), neuropilin-2 (NRP2), and EGFR—to mediate viral entry. These interactions activate the PI3K/AKT signaling pathway [15]. Following HCMV entry, the immediate early protein IE1 enhances the stem-like properties of GB cells by modulating cell-cycle progression and promoting survival in vivo [16]. IE1 also contributes to angiogenesis and loss of tumor-suppressive functions through downregulation of TSP-1 and GFAP [17], while also driving PI3K/AKT activation, reducing Rb phosphorylation, and inhibiting p53 signaling via decreased p21 expression [18,19]. These effects collectively enhance the proliferation and invasiveness of GB cells [20]. Consistent with this, RNA-seq analyses of patient-derived GB cells infected with HCMV revealed upregulation of GB-associated genes, including strong induction of c-MET via NF-κB activation, which has been linked to accelerated tumor growth [21].

Moreover, HCMV encodes a wide array of viral microRNAs (miRNAs) [22]. Among them, miR-UL112-3p, markedly upregulated in GB cells, targets TUSC3, a cellular mRNA, thereby stimulating AKT signaling pathway and promoting migration, invasion, cellular proliferation and overall poor prognosis and decreased patient survival [23]. In addition to encoding viral miRNAs, HCMV also modulates host cell miRNA expression. For instance, infection downregulates miR-134-5p in GB cells, resulting in increased levels of the anti-apoptotic factor ATF5 [24,25] and enhanced proliferative capacity [26].

Beyond miRNA, viral proteins also contribute to GB progression. The viral chemokine receptor homologue US28, expressed in approximately 60% of GB, enhances tumor invasion and promotes vascularization through VEGF induction [27,28] and IL-6/STAT3 and NFκB pathways [28,29]. US28 signaling also results in activation of the HIF-1/PKM2 [30]. HCMV also appears to modulate immune evasion mechanisms in GB through viral gene *UL111A*, an IL-10 homologue that suppresses proinflammatory cytokine production and downregulates MHC expression [31]. In addition, HCMV infection has been reported to enhance TERT activity to induce fibroblast survival [32]. HCMV can modulate both innate and adaptive immunity and reprogram cellular metabolism, collectively promoting an immunosuppressive tumor microenvironment. The virus expands populations of immunosuppressive cells, including Tregs and myeloid-derived suppressor cells (MDSCs), induces pro-tumoral cytokines, and upregulates GLUT1 and MCT4, driving lactate production that enhances angiogenesis, suppresses immune responses, and alters epigenetic states in neighboring non-infected cells, thereby contributing to GB progression [33,34]. HCMV also impairs NK cell recognition and cytotoxicity, disrupts dendritic cell maturation and antigen presentation, and promotes further Treg expansion, collectively dampening anti-tumor immunity [35,36,37]. These combined effects create an immunosuppressive microenvironment that facilitates GB growth. HCMV infection has further been shown to activate JNK signaling and induce morphological changes in GB cells, including reduced expression of E-cadherin and increased expression of vimentin [38]. These alterations result in loss of cell polarity and an epithelial-to-mesenchymal-like transition, favoring enhanced migration and invasiveness [39]. Collectively, these findings highlight the multifaceted role of HCMV in GB progression through the regulation of apoptosis, oncogenic signaling, immune evasion, metabolic reprogramming, and invasive potential.

Nevertheless, studies suggest that HCMV may function not only as an oncomodulator but also as a direct reprogramming vector. Recently, it has been shown that the infection of human astrocytes with clinical isolates of HCMV from GB patient biopsies induces the formation of GB-like cells. These cells possessed characteristics that mirror key features of aggressive GB, such as dedifferentiation and stemness, accompanied by an elevated expression of Myc and EZH2, elevated proneural–mesenchymal transition (PMT) markers, spheroid formation and invasiveness [40]. This ability to induce the transformation of cells has also been demonstrated in human embryonal lung fibroblasts and human mammary epithelial cells in vitro [41,42]. A limitation of these studies, however, is that they were conducted primarily at the cellular level and in patient biopsy samples, without validation in a relevant in vivo model.

Moreover, xenografting of HCMV-induced GB spheroids, generated from HCMV-infected human astrocytes, into immunodeficient Ragγ2C−/− mice resulted in the formation of tumors with a GB-like phenotype. These HCMV-derived tumors were characterized by glial proliferation, nuclear atypia with enlarged hyperchromatic nuclei, and high nestin expression, recapitulating key histopathological features observed in patient-derived GB xenografts. In addition, the TME of HCMV-derived tumors exhibited elevated Myc and EZH2 expression, paralleling findings in GB patient biopsies. Importantly, the presence of HCMV genes (*IE1* and *UL69*) and proteins (IE1 and IE2) was consistently detected in these tumors even two months post-engraftment, suggesting a sustained association between HCMV and tumor progression. Finally, elevated levels of HCMV, Myc, EZH2, and EGFR were also observed in the patient-derived model (PDX-P3), a well-characterized GB positive control. These findings further raise questions about the potential contribution of HCMV to GB initiation and progression in patient-derived models [43].

## 5. HCMV as a Therapeutic Target in GB

HCMV has emerged as a highly promising therapeutic target in GB, given its detection in tumor tissues and its influence on both tumor progression and the immune microenvironment (Table 2).

As previously described, HCMV promotes a tumor-supportive microenvironment and may contribute to therapy resistance. Consequently, targeting HCMV may enhance the efficacy of conventional GB treatments. Current research is exploring both the direct inhibition of HCMV and its viral proteins, as well as the use of HCMV antigens in immunotherapeutic approaches. The role of several viral proteins in tumor progression further underscores HCMV as a critical therapeutic target, fueling the development of novel treatment strategies. Peptide-based vaccination strategies targeting HCMV antigens have gained increasing attention in the context of GB. Vaccines incorporating a mutated form of the HCMV immediate early protein (HCMV-IE1mut) when combined with tumor-associated antigens such as Fibrinogen-like protein 2 (FGL2) have demonstrated synergistic effects in preclinical models. This combinatorial approach not only enhanced antitumor activity but also promoted stronger and more durable immune responses, highlighting its potential as a novel immunotherapeutic strategy against GB [44].

Another strategy consists of blocking viral entry receptors. The ephrin type A receptor 2 (EphA2), a member of the receptor tyrosine kinase family, is frequently overexpressed in GB. EphA2 has been identified as a critical host factor that mediates HCMV entry and membrane fusion in GB cells, thereby facilitating HCMV infection. Pharmacological or genetic inhibition of EphA2 has been shown to reduce HCMV infection and to attenuate tumor progression, highlighting its potential as a therapeutic target [46]. MicroRNA-based therapy can also be used to modulate microRNAs. Topoisomerase II alpha (TOP2A), which is silenced by microRNAs such as miR-139 and miR-548c-3p, plays a critical role in glioma cell proliferation and migration [49,50]. miR-144-3p, a member of the miR-144/451 cluster, acts as a tumor suppressor by directly targeting TOP2A, which is overexpressed in HCMV-positive gliomas. Restoration of miR-144-3p expression inhibits glioma growth in xenograft models, indicating that the upregulation of miR-144-3p can suppress the proliferation of HCMV-positive GB cells through inhibition of the TOP2A pathway [47]. Moreover, as previously described, the HCMV-encoded chemokine receptor US28 further contributes to tumor progression by activating intracellular signaling pathways. To counteract this effect, nanobodies specifically targeting US28 have been developed and shown to inhibit GB cell growth in vitro and in vivo. These nanobodies exhibit significant diagnostic and therapeutic potential for HCMV-associated GB, as preclinical mouse models demonstrate their efficacy in suppressing tumor growth [45].

To date, the majority of available HCMV therapies—(GCV, Cymevene R ©), valganciclovir (VGCV, Valcyte R ©), cidofovir (CDV, Vistide R ©) and foscarnet (FOS, Foscavir R ©)—act by interfering with the activity of the viral DNA polymerase pUL54. Interestingly, conventional antivirals such as valganciclovir, which target active viral replication, have been linked to improved survival outcomes in both non-randomized and randomized clinical studies [14,48]. Despite encouraging preclinical evidence, clinical translation remains limited, mainly due to the absence of standardized methods for detecting HCMV in GB and the paucity of large randomized trials. Notably, an ongoing multicenter phase 2 randomized trial (NCT04116411) is currently assessing the addition of valganciclovir versus placebo to standard therapy (radiotherapy plus temozolomide) in adults with newly diagnosed GB. However, these drugs are associated with significant toxicity and have contributed to the emergence of multiple drug-resistant HCMV strains [6]. In recent years, two novel compounds—maribavir and letermovir—have been introduced, targeting the viral kinase pUL97 and the terminase complex (pUL51-pUL56-pUL89), respectively. Although these newer agents demonstrate improved safety profiles, the rapid emergence of resistance remains a significant challenge [6,51]. New therapeutic targets are currently under investigation. Among the potential candidates, the terminase complex, which plays a key role in viral DNA packaging, stands out. Even if letermovir succeeds in inhibiting the packaging step by acting on the terminase complex, further studies could identify new functional motifs essential to its activity [52,53,54,55]. Another promising target is the helicase-primase complex, which is central to viral DNA replication by unwinding the DNA and synthesizing RNA primers required for DNA polymerase function. Notably, this complex is already effectively targeted in other herpesviruses such as HSV-1, HSV-2, and VZV [56]. However, none of the antivirals currently available are effective against the helicase-primase complex of HCMV. It is therefore crucial to characterize this complex to develop new therapeutic alternatives.

HCMV represents a promising therapeutic target in GB, with experimental evidence indicating that it influences tumor biology via complex oncomodulatory mechanisms. Nevertheless, methodological and clinical uncertainties remain. Progress in HCMV-targeted therapies will depend on standardized detection methods, a more comprehensive understanding of virus–tumor interactions, and rigorous validation in well-designed clinical trials.

## 6. Future Directions and Perspectives

The detection of HCMV in GB has produced highly inconsistent results, likely due to differences in detection methodologies, the specificity and sensitivity of the antibodies used, tissue processing and preservation procedures, variations in viral load, and the interpretative criteria applied across studies. Despite this heterogeneity, the possibility that HCMV contributes to GB biology has prompted interest in targeting the virus, and anti-HCMV therapy has emerged as a promising strategy to slow GB progression. Preclinical studies and early clinical trials with valganciclovir have yielded encouraging results, warranting rigorous clinical validation. However, current anti-HCMV agents routinely used for prevention and treatment targeting the viral polymerase are associated with hematologic and renal toxicities, and resistance mutations have also been reported for all antivirals whatever the target [57]. Alternative strategies must therefore be developed. Recently, we provided the first proof-of-concept that peptides designed to disrupt the pUL56–pUL89 interaction can effectively inhibit HCMV replication [58]. This strategy offers a novel approach to complement or replace traditional antivirals, which are often limited by side effects and resistance issues. Overall, targeting HCMV in GB emerges as a highly promising strategy, uniting viral biology, immunotherapy, and cancer signaling into a coherent therapeutic framework. By complementing existing treatments, this approach holds real potential to advance personalized, more effective interventions for GB patients.

## Figures and Tables

**Table 1:** Viral and cellular markers involved in GB progression (Figure 1 related table).

Figure 1 Related Number	HCMV Markers	Cellular Markers and Receptors	Action	Physiopathological Results
1	gB		PDGFRα activation → Akt signaling	Cell growth Invasive capacity of GB Cellular proliferation and migration
2	IE1		Inhibition of TSP-1 and GFAP Inhibition of p21 → overactivation of NF-κB pathway → Increased production of GLUT1 and MTC4 factors → lactate production Increase of Akt signaling	Cell survival Tumor progression Angiogenesis Angiogenesis Suppressed immune responses Altered epigenetic signatures GB progression Cell growth Invasive capacity of GB Proliferation and migration
3	US28		Activation of VEGF Increased HIF pathway signaling → production of antioxidant genes Increased NF-κB signaling (p50-p65/c-Rel) Increase in Akt signaling	Cell invasion Tumor vascularization Suppression of DNA repair pathways Cell proliferation Cell proliferation and invasion Cell growth Invasive capacity of GBM Proliferation and migration
4	UL111a		Inhibited MHC expression Increased STAT3 signaling	Inhibition of proinflammatory cytokine production
5	miR-UL112-3p		Inhibition of TUSC3 → Increased Akt signaling	Cell growth Invasive capacity of GB Proliferation and migration
6		miR-134-5p	Activation of ATF5 cell marker	Cell proliferation
7		IL-6	Increased STAT3 signaling	Inhibition of proinflammatory cytokines production
8		TLR	Increased NF-κB signaling	Cell proliferation and invasion
9		EGFR	Activation	Cell multiplication
10		CCL5	Increased viral protein US28 expression	Tumor vascularization Suppression of DNA repair pathways Cell proliferation and invasion
11		JNK pathway	Increasing of JNK gene production → Increased vimentin and reduced E-cadherin productions	Morphological change in GB cell Loss of cell polarity Mesenchymal transition Migration and invasion
12		PMT markers	Activation	Invasion Spheroid formation
13		NF KB pathway	Increased STAT3 signaling	Inhibition of proinflammatory cytokines production
14		TERT promoter	Activation	Cell survival
15		EZH2	Activation	

**Table 2:** HCMV-targeted strategies in GB models.

Strategy	Mechanism/Target	Key Findings	Citation
HCMV-IE1mut-FGL2 vaccine	Immune activation	Enhanced antitumor effect, reshaped TME	[44]
US28-targeting nanobodies	Viral protein US28	Inhibited tumor growth in vitro/in vivo	[45]
EphA2 inhibition	Viral entry receptor	Reduced HCMV infection in GB cells/organoids	[46]
miR-144-3p restoration	TOP2A oncogene	Suppressed proliferation, promoted apoptosis	[47]
Conventional antivirals	Viral replication	Improved survival	[13,48]

## Data Availability

The original contributions presented in this study are included in the article. Further inquiries can be directed to the corresponding author.
